# Influence of Blood Sampling Service Process Reengineering on Medical Services Supply: Quasi-Experimental Study

**DOI:** 10.2196/51412

**Published:** 2024-11-12

**Authors:** Wenmin Liao, Rong He, Zhonglian He, Nan Shi, Dan Li, Aihua Zhuang, Feng Gan, Ying Sun, Chaofeng Li

**Affiliations:** 1 State Key Laboratory of Oncology Guangdong Provincial Clinical Research Center for Cancer Sun Yat-sen University Cancer Center Guangzhou China

**Keywords:** process reengineering, blood sampling, hospital administration, medical informatics, digital health, patient experience

## Abstract

**Background:**

Tertiary hospitals in China are confronted with significant challenges due to limited spatial capacity and workforce constraints, leading to saturated allocation of medical resources and restricted growth in medical service provision. The incorporation of digital health into medical service process reengineering (MSPR) marks a pivotal transformation and restructuring of conventional health service delivery models. Specifically, the application of MSPR to blood sampling services processes reengineering (BSSPR) holds promise for substantially enhancing the efficiency and quality of medical services through streamlining and optimizing these procedures. However, the comprehensive impact of BSSPR has been infrequently quantified in existing research.

**Objective:**

This study aims to investigate the influence of BSSPR on the efficiency and quality of medical services and to elucidate the key informative technological support points underpinning BSSPR.

**Methods:**

Data were collected from both the new and old laboratory information systems from August 1, 2019, to December 31, 2021. A combination of statistical description, chi-square test, and *t* test was used to compare check-in time and waiting time of outpatients before and after the implementation of BSSPR. An interrupted time-series design was used to analyze the impact of BSSPR on medical service efficiency and quality, enabling the control of confounding variables, including changes in medical human resources and both long- and short-term temporal trends.

**Results:**

BSSPR had an impact on the efficiency and quality of medical services. Notably, there was a significant increase in the number of patients receiving blood sampling services, with a daily service volume increase of ~150 individuals (*P*=.04). The average waiting time for patients decreased substantially from 29 (SD 36) to 11 (SD 11) minutes, indicating a marked improvement in patient experience. During the peak period, the number of patients receiving blood sampling services per working hour statistically increased from 9.56 to 16.77 (*P*<.001). The interrupted time-series model results demonstrated a reduction in patients’ waiting time by an average of 26.1 (SD 3.8; 95% CI –33.64 to –18.57) minutes. Although there was an initial decline in the number of outpatients admitted following BSSPR implementation, an upward trend was observed over time (β=1.13, 95% CI 0.91-1.36).

**Conclusions:**

BSSPR implementation for outpatients not only reduced waiting time and improved patients’ experience but also augmented the hospital’s capacity to provide medical services. This study’s findings offer valuable insights into the potential advantages of BSSPR and underscore the significance of harnessing digital technologies to optimize medical service processes. This research serves as a foundational basis and provides scientific support for the promotion and application of BSSPR in other health care contexts. By continuing to explore and refine the integration of digital technologies in health care, we can further enhance patient outcomes and elevate the overall quality of medical services.

## Introduction

Optimizing medical services to provide faster, superior care that benefits a broader populace has consistently been the focus point of every health system, with this need becoming increasingly urgent in the post–COVID-19 era globally [1]. Three years into the COVID-19 pandemic, over 765 million confirmed cases [2] have persistently strained health systems, exacerbating the global medical supply-demand imbalance [3]. Drawing from years of pandemic response experience, medical institutions must not only deliver health services but also prevent cross-infections to ensure patients’ safety [4,5], which has posed a huge challenge for the Chinese medical system given its vast population and limited medical resources. Since the 2009 medical reform, the overall efficiency of national medical resources has increased, yet most provinces have entered the stage of diminishing returns in the medical resource allocation scale [6]. Therefore, enhancing technical efficiency and promoting total factor productivity changes with technological advancement is of great practical significance in creating a secure, orderly, and efficient medical environment in the new context [7].

Due to spatial constraints and workforce limitations, tertiary hospitals face challenges related to the saturated allocation of medical resources and limited growth of medical services [8]. According to the China Health Statistical Yearbook 2020, there were 954,390 primary-level medical institutions in China, accounting for only 32.6% of patient visits, whereas 2,749 tertiary hospitals accounted for 35.5% of patient visits [9]. This conflict is particularly acute in tertiary cancer hospitals, where it is estimated that over 4.5 million new cancer cases and more than 3 million cancer-related deaths were diagnosed in China in 2020 [10]. Given the complexity of tumor treatment, it is not appropriate to use hierarchical diagnosis and treatment policy to divert patients.

In the face of these challenges, business process reengineering emerges as a potent strategy to achieve dramatic productivity improvements without compromising the quality and scope of core health care services [11]. Technological innovation through the “internet plus health care” paradigm [12], also known as digital health [13], can achieve the goal of providing high-quality medical services to more patients with limited resources. In this context, medical service process reengineering (MSPR) supported by digital health represents a fundamental thinking and reconstruction of the hospital’s business process, aiming for overall improvement of cost, quality, service, and efficiency, and fostering a modern patient-centered medical environment.

**Figure 1:**
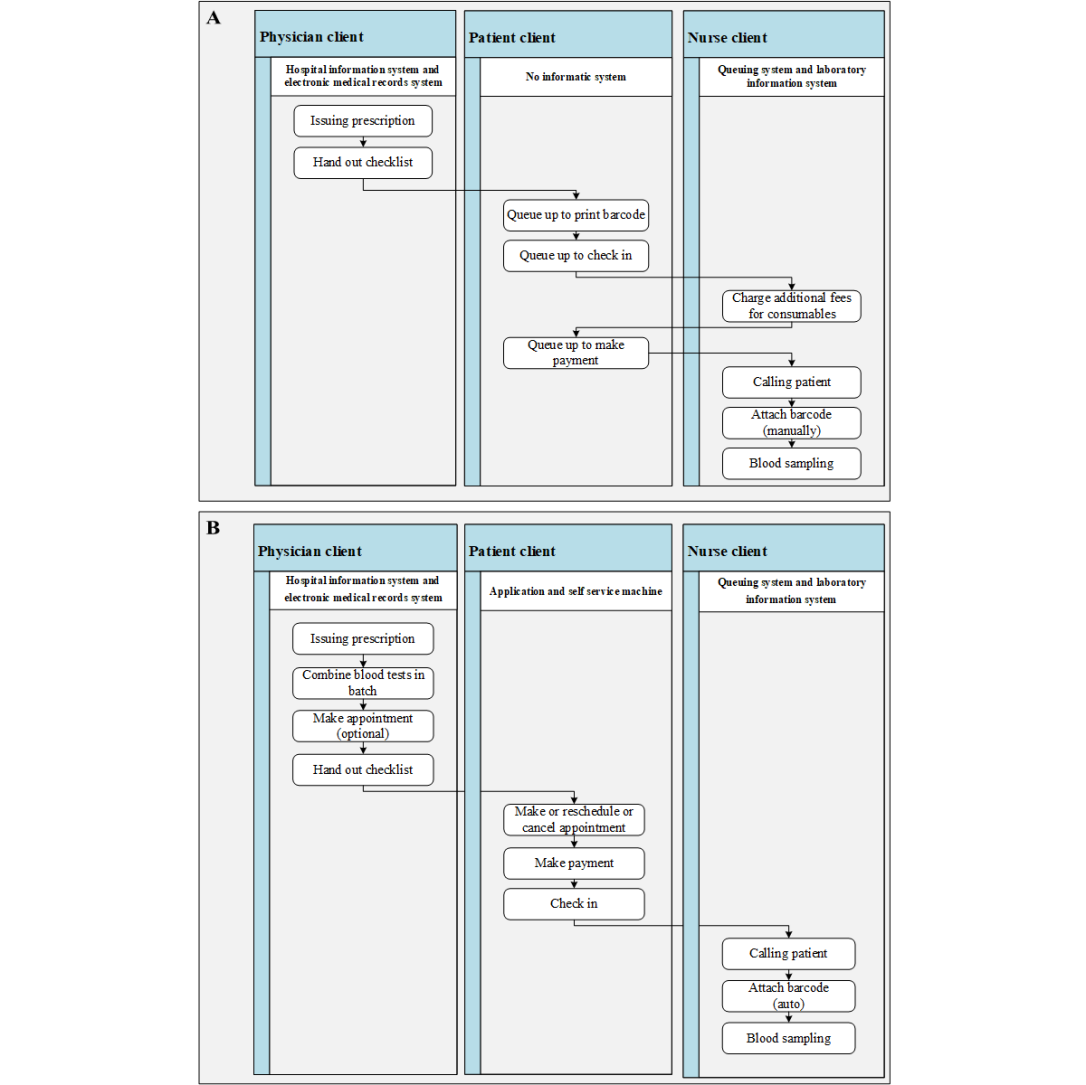
Blood sampling service process of outpatients (A) before and (B) after blood sampling service process reengineering.

In this study, a case of MSPR from a tertiary cancer hospital in Guangzhou, China, was presented. The Sun Yat-sen University Cancer Center (SYSUCC) is one of the oncology hospitals with the largest scale and the strongest academic strength in China, which integrates medical treatment, teaching, scientific research, and prevention. In 2020, SYSUCC had approximately 5000 outpatient visits daily, with an average of over 1000 daily blood tests. Before the reengineering of the blood sampling process, patients were required to queue up at the blood sampling center to print their individual barcodes after a doctor had prescribed a blood test. Once the barcode was printed, patients would need to queue up again for the actual blood sampling procedure. In cases where patients were prescribed multiple blood tests during a single clinic visit, they would take the centrally printed barcodes home for safekeeping and bring them back for their subsequent visit. The nurses would charge patients additional fees for consumables based on the different combinations of samples collected (Figure 1A). Consequently, the blood sampling center frequently experienced long queues, chaotic scenes, charge errors, and barcode allocation errors during peak hours.

Confronted with the challenges of high patient volume and limited service space, the blood sampling service process reengineering (BSSPR) was conducted in SUSYCC, supported by the Department of Information Technology. The BSSPR primarily encompassed the following aspects (Figure 1B): first, considering periodic blood sampling and testing needs of patients with cancer, doctors were required to batch blood tests after issuing prescriptions, with the system supporting batch appointment scheduling. Second, the system enabled appointment scheduling with a granularity of half an hour. Appointments can be made by doctors and patients through mobile apps or self-service machines, all of which could be modified at any time before the appointed day. Finally, a queuing and calling process was established within the blood sampling center: during the corresponding appointment period, patients could check in using mobile apps or self-service machines and await their call. Upon checking in, the blood sampling equipment would automatically standby with the barcode attached, allowing the nurse to sample blood immediately after calling.

The interrupted time-series (ITS) design is recognized as one of the most potent quasi-experimental designs, quantifying the effect of an intervention by comparing data before and after the intervention [14]. ITS necessitates a clearly defined intervention implementation timepoint and data collection at multiple equally spaced time points before and after the intervention (eg, weekly, monthly, or annually). By modeling preinterruption data to predict postinterruption outcomes and offering a counterfactual scenario for what would have transpired if the interruption had not occurred, akin to a comparator group in a randomized trial, ITS provides a range of effect estimates to describe the intervention’s impact. For instance, a level change corresponds to the difference between the time point of interest and the predicted preintervention level, while a slope change corresponds to the difference between the postintervention trend and the preintervention trend [15,16]. In recent years, ITS has been extensively used to measure the effectiveness of health care policies and programs [17,18].

With BSSPR as the study event, this research aims to use the ITS design to explore the impact of MSPR on the efficiency or quality of medical services and discuss the key elements of successful MSPR implementation, thereby providing practical construction experience.

## Methods

### Research Data

Treating the BSSPR, which finished on August 1, 2020, in SYSYCC as an interest, for comparative analysis, data from the preceding and subsequent laboratory information system (LIS) were used. Specifically, data from the old LIS, representing the period before BSSPR, spanning from August 1, 2019, to October 30, 2020, were compared with those from the new LIS, reflecting the post-BSSPR era from September 28, 2020, to December 31, 2021. The old LIS dataset comprised anonymized patient names, medical record numbers, barcode numbers, barcode printing time, sampling time, sampling nurses, and blood test items. For the old LIS dataset, due to the lack of a reservation mechanism, the accurate check-in time of patients cannot be obtained; therefore, the barcode printing time is used as the check-in time of patients. Therefore, the waiting time was the difference between sampling time and barcode printing time. Conversely, the new LIS dataset included the same patient identifiers and barcode number but substituted barcode printing time with appointment and check-in times, thereby facilitating the calculation of waiting times after BSSPR. Specifically, the patient’s waiting time is now the difference between sampling time and check-in time.

Initially, a comprehensive dataset encompassing 901,730 patient records was assembled. However, rigorous data cleaning was conducted to ensure analytical rigor. Records of 125,397 individuals were excluded from the old LIS analysis due to time differences exceeding 1 day between sampling and barcode printing, which indicated potential barcode reuse from previous visits. In addition, 60,654 individuals undergoing the blood routine test project were omitted from both LIS datasets, as the hospital designated a dedicated window for this project, negating the need for appointments. Furthermore, 32,064 records pertaining to nonworking hours (before 6 AM or after 5:30 PM on weekdays, and all day on weekends) were excluded from both datasets to maintain temporal consistency. Consequently, the final analysis encompassed 683,615 blood collection records from patients visiting between 6 AM and 5:30 PM on weekdays during the study period.

### Quantitative Analysis

The distribution of check-in time and waiting time of patients in the new and old LIS were statistically described. According to the check-in time distribution of outpatients, the peak period was set from 7 AM to 10 AM every day, and the rest was set as an off-peak period.

A series of two-tailed *t* test comparisons were used to compare the overall work efficiency per working hour before and after the implementation of the BSSPR, specifically assessing the average number of blood sampling tubes and patient encounters per hour per nurse. Comparisons were conducted separately for all day, peak hours, and off-peak hours, illuminating the effects of BSSPR on blood sampling efficiency across different time periods. Chi-square tests were used to compare the distribution of patient check-in time within 1 day and 1 week before and after BSSPR, respectively, to quantify the impact of BSSPR on the number of patients received.

ITS design [15] was used to analyze the impact of the BSSPR on the quantity and speed of medical services provision under the condition of controlling changes in medical human resources and long- and short-term time trends. The model framework is as follows:

y_ct_ = a + β_1_T_ct_ + β_2_Trend_t_ + β_3_T_c_ × Trend_t_ + β_4_wday_t_ + β_5_workforce_t_ + ε

Wherein, the dependent variables *y_ct_* are the daily total number of outpatients (Model 1) and the daily average waiting time of outpatients (Model 2) at *day_t_*, respectively; *T_ct_* is a dummy variable indicating before (coding as 0) and after (coding as 1) the BSSPR at *day_t_*, which makes *_1_* the post-BSSPR level change of dependent variables; *Trend_t_* is the count of days from August 1, 2019, to *day_t_*, which makes *_2_* the natural trend of dependent variables by day, used to control long-term time trends; and *_3_* indicates the post-BSSPR trend change of dependent variables. The temporal patterns (short-term time trend) were controlled using *wday_t_*, indicating Monday to Friday, and medical human resource change was controlled by *workforce_t_*, which denotes the total daily working hours of nurses at *day_t_*. The authors used the unique identifiers of sampling nurses in the dataset to determine whether a nurse was engaged in blood sampling activities every half-hour and calculated the total amount of health care workforce (in working hours) dedicated to blood sampling each day.

R (version 4.0.2, R Development Core Team) was used to conduct all statistical analysis, with packages *plyr*, *stringr*, *dplyr*, *lubridate*, *doBy*, *ggplot2*, *ggtext*, and *Epi*.

### Ethical Considerations

The study was granted ethical approval by the ethics committee of SYSUCC (B2023-486-01). Given the retrospective nature of the study, the requirement for informed consent was waived by the respective boards. Rigorous measures were put in place to anonymize all individual data, including the removal of identifiers like names, patient IDs, and contact details. Access to the data was strictly limited to authorized personnel, and it was stored securely to prevent any unauthorized access or disclosure, ensuring privacy protection while facilitating population-level analysis. No identification of individual participants or users was possible in any images of the paper. No financial or other forms of compensation were provided to participants in this study, as it was a retrospective analysis of existing data.

## Results

### Influence of Outpatient BSSPR on the Volume of Medical Service Provision

**Figure 2:**
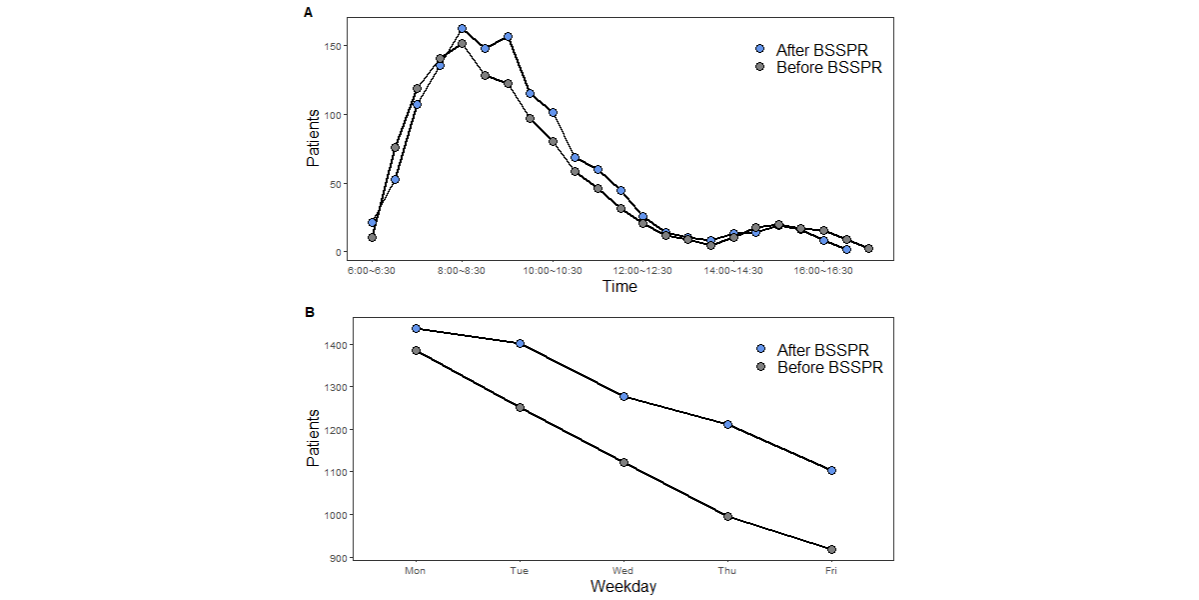
Distribution of outpatients’ check-in time before and after blood sampling service process reengineering (BSSPR): (A) daily pattern and (B) weekly pattern.

During the study period, a total of 683,615 patients underwent blood sampling, including 345,649 patients before BSSPR (1133 cases per day) and 337,966 patients after BSSPR (1285 cases per day). As shown in Figure 2, after BSSPR, the daily pattern of outpatients’ visiting was the same as that before BSSPR, and the peak appeared from 7 AM to 10 AM. However, after BSSPR, the average number of outpatients who checked-in during peak hours increased significantly (*P*<.001), of whom, the average number of outpatients who checked-in from 8:30 AM to 9 AM and 9 AM to 9:30 AM were 147 (SD 35) and 157 (SD 45), respectively, while before BSSPR, the corresponding number of admitted outpatients were 128 and 121, respectively. The chi-square (*χ*^2^) test showed a significant difference in the daily distribution of check-in time before and after BSSPR (*χ*^2^=130.67; *P*<.001). Analysis of the distribution of outpatients on weekdays showed that by using the old blood sampling service process, the number of outpatients admitted showed a continuous downward trend from an average of 1384 (SD 504) outpatients on Monday to an average of 919 (SD 370) outpatients on Friday. After BSSPR, not only did the average number of outpatients admitted on each Monday reach 1437 (SD 377) but also the numbers on Thursday and Friday were significantly increased compared with those before BSSPR, up to more than 1100 (*P*=.04). In general, after BSSPR, the number of admitted outpatients of blood sampling centers increased significantly (*χ*^2^=9.92; *P*=.04).

### Comparative Analysis of Blood Sampling Efficiency Before and After BSSPR

**Figure 3:**
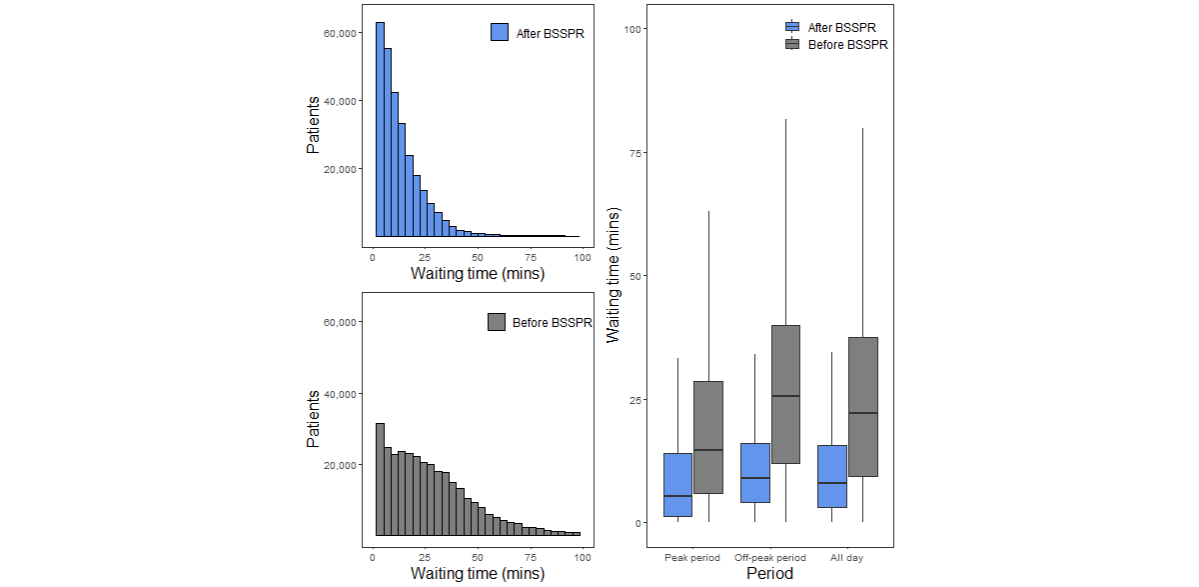
Distribution of outpatients’ waiting time before and after blood sampling service process reengineering (BSSPR).

The analysis of outpatients’ waiting time before and after the BSSPR showed that before BSSPR, the average waiting time of patients in the whole day, peak period, and off-peak period was 29 (SD 36) minutes, 30 (SD 36) minutes, and 26 (SD 38) minutes, respectively. After BSSPR, the patients’ average waiting time was 11 (SD 11) minutes, 11 (SD 10) minutes, and 10 (SD 13) minutes, respectively. As shown in Figure 3, after BSSPR, outpatients’ waiting time was significantly reduced in all periods and remained stable across peak period and off-peak periods, yet the waiting time of outpatients in peak period increased sharply before BSSPR.

Table 1 shows the influence of the BSSPR on blood sampling efficiency; the number of sampling tubes per hour of the whole day and peak period before and after BSSPR were not significantly different (all *P*>.05), yet the average number of sampling tubes per hour of the off-peak period was 23.47 (SD 18.47) and 33.36 (SD 11.32), before and after BSSPR, respectively, and the difference is statistically significant (*P*<.001). By comparing the number of outpatients per hour, it can be found that, before and after BSSPR, the average number of patients admitted per hour was 9.56 (SD 7.67) and 16.77 (SD 4.11) in the whole day, respectively, and the difference was statistically significant (*P*<.001). Statistically significant differences were also shown in the peak period (mean 10.52, SD 8.93 vs 19.60, SD 4.62; *P*<.001) and the off-peak period (mean 7.19, SD 6.56 vs 12.08, SD 5.64; *P*<.001). In the peak period and off-peak period, the number of admitted outpatients per work hour increased by 86% (9.08/10.52) and 68% (4.89/7.19) as a result of BSSPR.

**Table 1:** Comparative analysis on blood sampling efficiency.

Blood sampling efficiency		Before BSSPR^a^, mean (SD)	After BSSPR, mean (SD)	*t* test (*df*)	*P* value
**Sampling tubes (/working hour^b^)**					
	All day	45.69 (26.63)	43.70 (12.89)	–0.59 (187)	.55
	Peak period	51.52 (31.51)	53.12 (13.52)	0.40 (183)	.69
	Off-peak period	23.47 (18.47)	33.36 (11.32)	4.17 (225)	<.001
**Patients (/working hour)**					
	All day	9.56 (7.67)	16.77 (4.11)	7.35 (223)	<.001
	Peak period	10.52 (8.93)	19.60 (4.62)	7.98 (221)	<.001
	Off-peak period	7.19 (6.56)	12.08 (5.64)	5.73 (243)	<.001

^a^BSSPR: blood sampling service process reengineering.

^b^Working hour means 1 nurse working for 1 hour.

### Attributed Influence of BSSPR on the Speed and Volume of Outpatient Medical Service Provision

The results of the ITS regression model are shown in Table 2. Although the BSSPR resulted in an average decrease of 106.62 patients per day in the peak period, a set of slight but significant increasing trends of patient counts after BSSPR have been detected in the whole-day, peak, and off-peak periods (β=1.13, .88, and .21, respectively; all *P*<.001). The effect of increasing the number of working hours on the number of patients received was statistically significant in every period (all *P*<.001), and an increase of 1 working hour could increase the number of admitted outpatients on average by 13.15 (SD 0.15).

The BSSPR reduced the waiting time of patients by 26.1 (95% CI –33.64 to –18.57) minutes in total, within the peak time by 27.52 (95% CI –35.47 to –19.56) minutes, and within the off-peak time by 22.33 (95% CI –30.46 to –14.20) minutes. Patients’ waiting time was not significantly reduced by adding to the workforce (all *P*>.05). It has been detected that there is a very slight increasing trend in patients’ waiting time after BSSPR, as well as during the peak period (β=0.02).

**Table 2:** Attributed influence of BSSPR^a^ on the speed and volume of outpatient medical service provision.

Model			All day				Peak period					Off-peak period		
			β (95% CI)		*P* value	β (95% CI)			*P* value		β (95% CI)			*P* value
**Model 1^b^**														
	Change after BSSPR	–78.87 (–208.12 to 50.38)		.23		–106.62 (–199.56 to –13.69)		.02		45.44 (–7.96 to 98.83)			.10	
	Trend after BSSPR	1.13 (0.91 to 1.36)		<.001		0.88 (0.71 to 1.04)		<.001		0.21 (0.12 to 0.31)			<.001	
	Natural trend	–0.50 (–0.58 to –0.42)		<.001		–0.32 (–0.39 to –0.26)		<.001		–0.16 (–0.2 to –0.12)			<.001	
	Working hour	13.15 (12.87 to 13.44)		<.001		14.87 (14.52 to 15.23)		<.001		10.16 (9.84 to 10.48)			<.001	
**Weekday (Monday as reference)**														
	Tuesday	–43.08 (–71.42 to –14.74)		.003		–31.51 (–52.28 to –10.73)		.003		–10.24 (–29.18 to 8.71)			.29	
	Wednesday	–72.62 (–89.26 to –55.97)		<.001		–41.85 (–60.73 to –22.97)		<.001		–42.76 (–58.8 to –26.72)			<.001	
	Thursday	–121.32 (–145.16 to –97.48)		<.001		–71.22 (–91.52 to –50.92)		<.001		–48.82 (–64.33 to –33.3)			<.001	
	Friday	–118.82 (–138.36 to –99.28)		<.001		–80.27 (–101.41 to –59.13)		<.001		–44.43 (–60.19 to –28.67)			<.001	
**Model 2^c^**														
	Change after BSSPR	–26.10 (–33.64 to –18.57)		<.001		–27.52 (–35.47 to –19.56)		<.001		–22.33 (–30.46 to –14.20)			<.001	
	Trend after BSSPR	0.02 (0.00 to 0.03)		.04		0.02 (0.00 to 0.04)		.03		0.005 (–0.01 to 0.02)			.65	
	Natural trend	–0.01 (–0.02 to 0.01)		.21		–0.01 (–0.03 to 0.00)		.11		0.01 (–0.01 to 0.02)			.52	
	Working hour	–0.02 (–0.07 to 0.02)		.32		–0.05 (–0.13 to 0.03)		.26		–0.09 (–0.23 to 0.05)			.19	
**Weekday (Monday as reference)**														
	Tuesday	–3.98 (–7.06 to –0.90)		.01		–2.75 (–5.82 to 0.32)		.08		–6.68 (–10.54 to –2.82)			.001	
	Wednesday	–3.97 (–7.08 to –0.87)		.01		–1.52 (–4.68 to 1.64)		.35		–9.00 (–12.76 to –5.25)			<.001	
	Thursday	–3.09 (–6.26 to 0.08)		.06		–0.30 (–3.55 to 2.94)		.85		–9.26 (–13.00 to –5.51)			<.001	
	Friday	–1.40 (–4.72 to 1.91)		.41		1.37 (–2.01 to 4.76)		.43		–8.40 (–12.30 to –4.50)			<.001	

^a^BSSPR: blood sampling service process reengineering.

^b^The dependent variable is the daily total number of outpatients.

^c^The dependent variable is the daily average waiting time of outpatients.

## Discussion

### Principal Findings

This study took the BSSPR event of a tertiary oncology hospital in China as a focus and introduced the practice of MSPR through digital health. It was found that after BSSPR, the advanced management of patients’ flow was effective, and medical resources were used more efficiently during off-peak hours such as Friday. BSSPR has not only improved the capacity of medical institutions to provide medical services but also significantly reduced the waiting time of outpatients. These results indicated the feasibility and applicability of using digital health to optimize the medical service process in the face of the bottleneck of tertiary hospitals in improving their ability to provide medical services.

Through our work, we have outlined several of the cores of MSPR to provide some practical experience. First, a common way of MSPR is to make medical service provision in a planned and orderly way as far as possible, such as proactive and rational management of patient flow in advance [19]. In this way, it not only avoids the adverse impact of the rush hour crowd on the medical environment but also reduces the waste of medical resources during the nonrush hour, to maximize the use of medical resources. The realization of this in BSSPR described in this study depends on the establishment of a unified appointment platform: making an appointment for blood sampling from multiple channels, including an electronic medical records system (by doctors), mobile apps, or self-service machines (by patients); sharing the same appointment pool and the same queue; and allowing patients to make, reschedule, and cancel appointments within certain rules [20].

This approach greatly reduces the invalid waiting time of patients, thus creating a win-win situation, which not only avoids the adverse impact of long waiting time on patients’ satisfaction [21-23] and willingness to revisit [8,24] but also improves the use of health care services and guides the growth of hospital medical service [25]. Advanced management of patient flow may also make more effective use of medical resources in leisure time [26]. Before BSSPR, many patients would not choose Friday for blood tests, which may be due to the suspension of the clinic on weekends. In the absence of appointment rules, patients in other cities would consider it risky to see a doctor on Friday, because they would have to wait for a whole weekend if they missed it. After BSSPR, the number of outpatient visits on Friday increased significantly, confirming the previously stated patient concerns and the powerful effect of the establishment of appointment rules in BSSPR on the allocation of medical resources over time.

Second, the MSPR related to patient services needs to pay much attention to user-friendliness and message feedback mechanisms. After BSSPR, doctors would hand out the outpatient examination checklist with the appointment rules and matters needing attention after issuing the examination order, so as to improve the friendliness of the process of appointment and check-in to patients. At the same time, an instant message will be sent after making appointments and changing appointments through mobile apps or self-service machines. The convenience of self-service operation and message transmission makes patients more active in operation. Although the results of the ITS model showed a decrease in the number of outpatient patients received by blood sampling center during the peak period after the implementation of BSSPR, this may be due to the diversion of patients from the peak period to another period at the early stage of the system’s launch, which can also indicate that the medical resources of blood sampling center were overloaded before the implementation of MSPR. The upward trend in outpatient numbers after MSPR is implemented also shows that proper MSPR can improve hospital efficiency in the long run as patients adapt to the new service mode.

Third, the effectiveness of MSPR can be maximized only when MSPR is customized according to the characteristics of patients or diseases. In this case, according to the characteristics of patients with cancer undergoing periodic blood tests, the function of appointment by batch is realized. In other scenarios, for example, the return visit time of clinical research patients should be strictly calculated by the system, the follow-up visit time of postoperative patients should be personalized according to the follow-up results, and so on, and digital technology should be used to realize smart services.

Finally, leveraging detailed data recorded by the information system, we observed and analyzed service system operations to achieve lean management objectives, exemplifying this case’s practical implications. In the actual scenario, the waiting time can be estimated by the length of the queue, which is beneficial for patients to choose the optimal route [27]. The calculated increase in the number of patients received and the decrease in waiting time due to the improvement of human resources can be used to flexibly allocate the number of nurses and equipment according to the immediate waiting time and the number of queuing people, so as to reduce the redundancy and shortage during peak hours [28]. In addition, calculating the number of visits and productivity of each nurse facilitates more accurate performance evaluations. Therefore, the optimal situation of patients, medical resource allocation, and service management can be achieved.

The novelty of this paper resides in its employment of an ITS design to appraise the impact of an MSPR initiative. By controlling both short- and long-term trends alongside health care human resource variations, this methodology enables the analysis of longitudinal administrative data, offering a robust retrospective assessment of digital technologies’ efficacy within health care settings [29]. Moreover, our contribution extends to a comprehensive discourse on the pivotal steps involved in medical process reengineering.

However, the study had some limitations. First, the study’s limitation primarily stems from its focused scope, encompassing a specific health care context and MSPR endeavor. As a single-center study, the findings’ generalizability to diverse health care settings or interventions remains uncertain. Furthermore, the study’s reliance on retrospective data analysis and ITS design may be constrained by inherent limitations, including data incompleteness or potential biases. An expanded exploration of confounding variables potentially influencing outcomes could have further strengthened the study. Nevertheless, this research presents valuable insights, underscoring the need for future endeavors to address the identified limitations and build upon our findings.

### Conclusions

The implementation of BSSPR, an example of MSPR in a tertiary cancer hospital in China, not only improved the capacity of medical institutions to provide medical services but also significantly reduced the waiting time of outpatients. This provides a model for using digital technology in MSPR. Future research should focus on providing an evaluation model for digital MSPR to guide the efficient application of information technology in improving the efficiency of medical resources.

## Abbreviations

BSSPR: blood sampling service process reengineering
ITS: interrupted time-series
LIS: laboratory information system
MSPR: medical service process reengineering
SYSUCC: Sun Yat-sen University Cancer Center

## Data Availability

The datasets generated during and/or analyzed during this study are not publicly available due to their inclusion of sensitive hospital business operational data but are available from the corresponding author on reasonable request.
